# Welche Bedarfe haben Studierende hinsichtlich gesundheitsförderlicher Maßnahmen und welche sind ihnen bekannt? Eine Befragung an zwei Hochschulstandorten

**DOI:** 10.1007/s11553-023-01031-w

**Published:** 2023-04-25

**Authors:** Roxana Schweighart, Jessica Thätz, Lisa Demar, Franziska Zehl, Silke Neuderth, Rebecca Löbmann

**Affiliations:** Institut für Angewandte Sozialwissenschaften, Hochschule für Angewandte Wissenschaften Würzburg-Schweinfurt, Würzburg, Deutschland

**Keywords:** Studentisches Gesundheitsmanagement, Stress, Psychische Gesundheit, Prävention, Universität, Student health management, Stress, Mental health, Prevention, University

## Abstract

**Hintergrund:**

Viele Studierende in Deutschland sind von gesundheitlichen Beeinträchtigungen, darunter primär von Stress und psychischen Belastungen, betroffen. Die Prävalenz psychischer Störungen nimmt unter Studierenden kontinuierlich zu. Im Rahmen des Studentischen Gesundheitsmanagements an der Hochschule für Angewandte Wissenschaften Würzburg-Schweinfurt wurden Bedarfe und Wünsche zu Gesundheitsbereichen, die Studierende als relevant für ihr Studium betrachten, erfasst und das Wissen über vorhandene Gesundheitsangebote ermittelt.

**Methoden:**

Im Mai 2022 wurde ein standardisierter Online-Fragebogen per E‑Mail an alle Studierenden der Hochschule Würzburg-Schweinfurt geschickt. Unterschiede zwischen den Hochschulstandorten sowie zwischen deutsch- und nicht-deutschmuttersprachigen Studierenden wurden über nonparametrische Verfahren ermittelt.

**Ergebnisse:**

Stress und psychische Belastung sind gesundheitsgefährdende Probleme, mit denen sich die Befragten besonders häufig konfrontiert sehen. Studieren in Schweinfurt und Fremdsprachlichkeit stehen in Zusammenhang mit einer erhöhten Relevanz einiger Gesundheitsbereiche. Unterstützungsangebote wünschen sich die Befragten v. a. in den Bereichen Sport/Bewegung, psychische Belastung/Erkrankung und Stressreduktion. Die Studierenden haben mitunter kaum Kenntnis über vorhandene Angebote. Vor allem Studierende in Schweinfurt und internationale Studierende sind kaum informiert.

**Schlussfolgerungen:**

Erstens sollten vorrangig Angebote zur Reduktion von Stress und psychischer Belastung an der Hochschule implementiert werden. Zweitens sollten zukünftig die gesundheitlichen Belange von Studierenden in Schweinfurt und internationalen Studierenden stärkere Beachtung finden. Drittens sollten alle Studierenden verstärkt über bereits bestehende Angebote informiert werden.

## Abstract (en)

**Background:**

Many students in Germany face health problems, primarily stress and psychological strain. The prevalence of mental disorders is continuously increasing among students. In the context of the student health management at the University of Applied Sciences Würzburg–Schweinfurt, we recorded needs and wishes regarding health areas that students consider to be relevant for their studies and we also assessed their knowledge about existing health offers.

**Methods:**

In May 2022, we sent a standardized online questionnaire via email to all students at the University of Applied Sciences Würzburg–Schweinfurt. Differences between university locations and between German- and non-German-native speaking students were determined using non-parametric tests.

**Results:**

Stress and psychological strain are health-threatening problems that respondents face particularly frequently. Studying in Schweinfurt and being a foreign language speaker relate to an increased relevance of some health areas. Respondents desire support especially in the areas of sports/exercise, mental stress/illness, and stress reduction. Students sometimes have little knowledge about existing offers. Especially students in Schweinfurt and international students are barely informed.

**Conclusions:**

First, offers to reduce stress and mental burden should be implemented at the university as a matter of priority. Second, the health concerns of students in Schweinfurt and international students should be given greater attention in the future. Third, all students should be informed more about existing offers.

## Hintergrund

Studierende sind nicht erst seit der COVID-19-Pandemie (coronavirus disease 2019) diversen Belastungen ausgesetzt. Dies zeigt eine von Herbst et al. [18] im Jahr 2016 durchgeführte Studie, bei der 18.000 in Deutschland Studierende befragt wurden. Laut den Ergebnissen liegt bei 53 % der Teilnehmenden ein hohes, bei 42 % ein mittleres und nur bei 5 % ein niedriges Stresslevel vor. Mitunter empfinden insbesondere Studentinnen, Studierende von Hochschulen für Angewandte Wissenschaften und Studierende der Fächer Veterinärmedizin, Agrar‑, Forst- und Ernährungswissenschaft, Informatik sowie Kunst und Kunstwissenschaft vermehrt Stress. Darüber hinaus sind Studierende stärker von Depressions- und Angstsymptomen betroffen als andere Bevölkerungsgruppen. So liegen bei 20 % der befragten Studierenden von vier Hochschulen Hinweise auf eine depressive Störung vor, während die Prävalenz für eine Angststörung je nach Hochschule zwischen 18 und 21 % schwankt [10]. Ähnliche Zahlen liefert der BARMER Arztreport von 2018 [16]. Laut dieser Erhebung wurde bei 17 % der bei der BARMER versicherten Studierenden mindestens eine psychische Erkrankung diagnostiziert. Im Vergleich dazu leidet laut sozioökonomischem Panel (SOEP) jede:r Zehnte in der deutschen Gesamtbevölkerung unter einer Depression oder Angsterkrankung [17]. Vergleicht man dagegen die Häufigkeiten von Diagnosen für psychische Erkrankungen zwischen Studierenden und jungen Erwerbstätigen, ist die Datenlage weniger eindeutig. Manche Studien kommen zu dem Schluss, dass Studierende nicht häufiger betroffen sind [16]. Andere Erhebungen legen jedoch nahe, dass Studierende durchaus häufiger als junge Erwerbstätige unter depressiven Episoden oder Angststörungen leiden und zudem des Öfteren Antidepressiva sowie andere Psychopharmaka verschrieben bekommen [15]. So ist z. B. laut einem Bericht der Techniker Krankenkasse die psychotherapeutische Behandlungsrate von Studierenden knapp doppelt so hoch wie die von jungen Erwerbstätigen [15]. Obgleich dieser Differenzen ist die Verbreitung psychischer Erkrankungen unter jungen Erwachsenen in den letzten Jahren insgesamt deutlich angestiegen. Von 2005 bis 2016 erhöhte sich der Anteil der 18- bis 25-Jährigen mit einer diagnostizierten psychischen Erkrankung um 38 %. Depressionen stiegen in diesem Zeitraum um 76 % an [16]. Dieser Trend ist auch unter Studierenden zu beobachten. Der Anteil der aufgrund depressiver Symptome behandelten Studierenden stieg zwischen 2004 und 2016 um 43 % von 2,7 auf 3,9 % [15]. Dabei scheint die COVID-19-Pandemie die (psychischen) Belastungen noch einmal zusätzlich verstärkt und die Gesundheit von Studierenden verschlechtert zu haben, wie mehrere Studien zeigen [9, 35]. Fast alle erhobenen Gesundheitsmaße (u. a. Gesundheitszustand, Burnout, Lebenszufriedenheit) fielen laut Umfrage unter Studierenden der Technischen Universität Kaiserslautern, im Sommersemester 2021 schlechter aus als noch im Jahr 2018 [5]. Eine Erklärung für die bereits vor der Pandemie besonders prekäre gesundheitliche Lage von Studierenden, liefert das systemische Anforderungs-Ressourcen-Modell (SAR-Modell) von Becker [4]. Dieses besagt, dass der Gesundheitszustand eines Menschen davon abhängt, wie gut er oder sie interne und externe Anforderungen bewältigen kann. Im Studium werden junge Erwachsene mit vielen neuen Anforderungen konfrontiert und müssen zuweilen erstmalig umfangreiche Eigenverantwortung übernehmen. Für die Bewältigung der im Studium anfallenden Anforderungen braucht es somit adäquate Ressourcen, deren Fehlen in einer schlechteren gesundheitlichen Verfassung der Studierenden resultieren kann. Im Umkehrschluss kann über die Anpassung der Anforderungen und/oder die Stärkung der individuellen Ressourcen die Gesundheitsförderung von Studierenden gelingen. Dieses Ziel im Blick, wurden für die vorliegende Studie Studierende der Hochschule für Angewandte Wissenschaften Würzburg-Schweinfurt (FHWS) zu ihren Bedarfen und Wünschen im Hinblick auf gesundheitsförderliche Maßnahmen befragt. Im Wintersemester 2021/2022 waren etwa 9300 Personen an dieser Hochschule eingeschrieben [20]. Eine Besonderheit der FHWS ist die Präsenz an zwei Standorten, die ca. 40 km voneinander entfernt liegen. Während die in Würzburg (WÜ) vertretenen Fakultäten von Natur- und Geistes-, über Sozial- bis hin zu Wirtschaftswissenschaften reichen, ist der Campus in Schweinfurt (SW) primär auf technische Studiengänge fokussiert. Dem größeren Studienangebot in WÜ entsprechend, sind dort zwei Drittel der an der FHWS Studierenden eingeschrieben. Der Standort in SW zeichnet sich hingegen durch einen großen Anteil internationaler Studierender aus, was sich durch das dortige Angebot sogenannter TWIN-Studiengänge in englischer Sprache erklären lässt [19].

Abgesehen davon unterscheiden sich die beiden Standorte bezüglich der vorhandenen Gesundheitsangebote für Studierende. Eine Bestandsanalyse aus dem Jahr 2020 konnte eine hohe Angebotsdichte an studierendenspezifischen Gesundheitsangeboten am Universitätsstandort WÜ feststellen [23]; dies dürfte v. a. durch vorhandene Institutionen wie Universitätsklinik, Hochschulsport und Sportzentrum, psychotherapeutische Ambulanzen, Studentenwerk und Katholische sowie Evangelische Hochschul- und Studierendengemeinden gewährleistet sein. SW ist hingegen industriell geprägt und zeichnet sich insgesamt durch eine geringere gesundheitsbezogene Infrastruktur aus, wobei nur ein einziges Angebot zielgruppenspezifisch für Studierende ausgelegt ist (interne Recherche durch den Bachelor Management im Gesundheitswesen im Jahr 2022). Zur Förderung der Gesundheit der Studierenden beider Standorte wird seit Anfang 2022 an der FHWS – gefördert durch die AOK Bayern – ein studentisches Gesundheitsmanagement (SGM) implementiert und evaluiert. Im Sinne einer standortspezifischen Bedarfserhebung wurde hierfür ermittelt, welche Gesundheitsbereiche die Studierenden als relevant für ihr Studium betrachten, welche Angebote sich Studierende wünschen, welche bereits bestehenden Angebote den Studierenden bekannt sind und, ob Unterschiede zwischen Hochschulstandort und Muttersprache hinsichtlich der untersuchten Variablen vorliegen. Die Antworten auf diese Fragen werden im vorliegenden Artikel präsentiert. Ferner wird diskutiert, inwiefern die gewonnenen Erkenntnisse für eine bedarfsorientierte Angebotsgestaltung im Rahmen des SGM genutzt werden können.

## Methode

Zur Beantwortung der genannten Forschungsfragen wurde ein querschnittliches Studiendesign gewählt. Im Mai 2022 wurde allen Studierenden der FHWS über den hochschulweiten E‑Mail-Verteiler ein Link zu einem standardisierten Online-Fragebogen gesendet. Die Datenerhebung wurde im Mai 2022 durchgeführt, um das laufende Semester zu umfassen, zugleich jedoch auch, um erhöhten Stress und vermehrte Belastungen während der Lern- und Prüfungsphase zu vermeiden. Der von den Studierenden auszufüllende Fragebogen wurde in deutscher und englischer Sprache angeboten und umfasste insgesamt 12 Fragen zu 6 Themengebieten: (1) soziodemographische Angaben, (2) relevante Gesundheitsbereiche, (3) gewünschte Angebote zur Gesundheitsförderung, (4) bevorzugte Sprechzeiten, (5) bevorzugte Form der Unterstützung (persönlich, telefonisch, über Zoom etc.) sowie (6) die Abfrage bekannter Angebote und Anlaufstellen in WÜ und SW. Die Studierenden wurden aufgefordert einzuschätzen, welche Relevanz die verschiedenen Gesundheitsbereiche in Bezug auf ihr Studium haben. Diese Einschätzung sollte anhand einer vierstufigen Skala (von „nicht relevant“ bis „sehr relevant“) vorgenommen werden. Bis auf diese und die Altersangabe (Angabe in Jahren) wurden alle weiteren Variablen nominal erfasst. Neben deskriptiven Auswertungen wurde aufgrund fehlender Normalverteilung und der Größenunterschiede der vorhandenen Teilstichproben zur Berechnung von Unterschieden bezüglich relevanter Gesundheitsbereiche ein nichtparametrisches Testverfahren (Mann-Whitney-U-Test) angewandt. Um herauszufinden, ob Studienort bzw. Fremdsprachlichkeit unabhängig von den gewünschten oder bereits bekannten Angeboten auftreten, wurden zusätzlich χ^2^-Tests berechnet. Die Auswertung der Daten erfolgte in SPSS [22].

## Ergebnisse

Insgesamt nahmen 511 Studierende an der Umfrage teil, wovon 65 % in WÜ studierten. Die Rücklaufquote betrug etwa 5,5 % und unterschied sich nicht signifikant zwischen den Standorten. Es nahmen Studierende aller zehn Fakultäten teil. Die Befragten waren zum Zeitpunkt der Befragung zwischen 18 und 63 Jahre alt, der Altersdurchschnitt lag bei 23,8 (Standardabweichung = 4,4) Jahren. 503 Personen machten valide Angaben zu ihrem Geschlecht, wovon sich 56 % als „weiblich“, 42 % als „männlich“ und < 1 % als „divers“ einordneten. 78 % der Studierenden gaben Deutsch als ihre Muttersprache an, während 20 % eine andere Muttersprache angaben. Hierfür lagen Angaben von 500 Befragten vor. Differenziert nach Hochschulstandort ließ sich feststellen, dass die Befragten des Standorts WÜ mehrheitlich weiblich waren (73 %) und fast durchweg Deutsch als Muttersprache sprachen (91 %). 73 % der Befragten aus SW waren hingegen männlich und knapp die Hälfte (48 %) nicht-deutschmuttersprachig.

### Relevante Gesundheitsbereiche

**Abb. 1:**
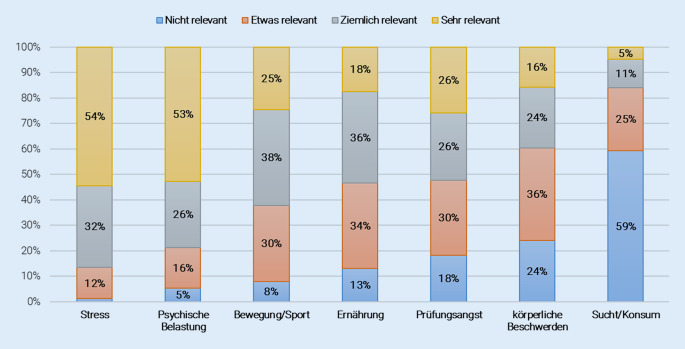
Relevante Gesundheitsbereiche

Unter allen Befragten (*n* = 511) waren vorrangig Stress und psychische Belastung von Bedeutung (Abb. 1). 86 % gaben an, dass Stress für sie persönlich im Hinblick auf das Studium sehr oder ziemlich relevant ist. Bezüglich der psychischen Belastung gaben dies mehr als drei Viertel der Befragten an (79 %). Sinkend in der Relevanz folgen Bewegung/Sport, Ernährung, Prüfungsangst, körperliche Beschwerden/Krankheit und abschließend Sucht/Konsum. Letzter Gesundheitsbereich war für mehr als die Hälfte nicht relevant in Bezug auf ihr Studium.

Für die Gesundheitsbereiche Prüfungsangst und körperliche Beschwerden/Krankheit zeigten sich signifikante Unterschiede zwischen den Hochschulorten. Für Studierende aus SW war Prüfungsangst signifikant relevanter (Median = 3; Q1 = 2; Q3 = 4) als für Studierende aus WÜ (Median = 2; Q1 = 2; Q3 = 3). Der Mann-Whitney-U-Test ergab für Prüfungsangst und Standort folgendes: U = 21.226,000, *p* < 0,001, r = 0,19. Die Effektstärke entspricht laut Cohen [8] einem schwachen Effekt (r < 0,3). Die Differenz in der Relevanz des Gesundheitsbereichs Prüfungsangst war zwischen den Standorten somit signifikant, jedoch eher gering. Auch körperliche Beschwerden/Krankheit waren für die Studierenden am Standort SW signifikant bedeutsamer (Median = 2; Q1 = 2; Q3 = 3) als für diejenigen, die in WÜ eingeschrieben waren (Median = 2; Q1 = 1; Q3 = 3). Der Mann-Whitney-U-Test ergab: U = 23.903,500, *p* = 0,010, r = 0,16. Somit ist hier ebenfalls ein schwacher Effekt feststellbar. Für die Bereiche Stress, psychische Belastung, Bewegung/Sport, Ernährung sowie Sucht/Konsum konnten keine signifikanten Unterschiede zwischen Studierenden der beiden Standorte festgestellt werden. Betrachtet man die Unterschiede hinsichtlich der Muttersprache, zeigten sich signifikante Unterschiede zwischen deutsch- und nicht-deutschmuttersprachigen Studierenden für die Gesundheitsbereiche Prüfungsangst, körperliche Beschwerden/Krankheit, Ernährung sowie Sucht/Konsum. In Tab. 1 ist der jeweilige Median, das erste und dritte Quartil, der Wert des Mann-Whitney-U-Tests (U), der Signifikanzwert (*p*) und die Effektstärke (r) abgebildet.

**Tab. 1:** Ergebnisse des Mann-Whitney-U-Tests für die signifikanten Gesundheitsbereiche in Zusammenhang mit der Muttersprache

		Prüfungsangst	Körperliche Beschwerden/Krankheit	Ernährung	Sucht/riskanter Konsum/Gebrauch
Median (Q1, Q3)	Deutsch	2 (2,3)	2 (1,3)	3 (2,3,)	1 (1,2)
	Andere	3 (3,4)	3 (2,3)	3 (2,4)	1 (1,3)
U		12.783,500	16.632,000	17.180,500	17.442,000
p		< 0,001	0,004	0,010	0,013
r		0,27	0,13	0,12	0,11

Alle in Tab. 1 präsentierten Bereiche wurden von den nicht-deutschmuttersprachigen Studierenden als relevanter eingestuft. Die Effekte sind nach Cohen [8] als schwach (r < 0,3) einzustufen. Für die Themen Stress, psychische Belastung und Bewegung/Sport konnten keine signifikanten Unterschiede zwischen den Studierenden unterschiedlicher Muttersprachen festgestellt werden.

### Gewünschte Gesundheitsangebote

Die Befragten konnten für neun Gesundheitsthemen angeben, ob sie in diesem Bereich ein Angebot an ihrem Hochschulstandort wünschen oder nicht. Unter allen Befragten lagen die meistgewünschten Angebote in den Bereichen Sport/Fitness/Bewegung (70 %), psychische Belastungen/Erkrankungen (69 %), Stressreduktion (65 %) sowie Entspannung/Achtsamkeit (50 %).

Zur Berechnung von Zusammenhängen zwischen Wunschangebot und Hochschulstandort bzw. Muttersprache wurden χ^2^-Tests durchgeführt. In Tab. 2 sind die gewünschten Angebote mit ihren Häufigkeiten, unterteilt nach Hochschulstandort und Muttersprache abgebildet.

**Tab. 2:** Häufigkeit der gewünschten Angebote differenziert nach Hochschulstandort und Muttersprache

Angebote gewünscht zu …	Standort				Muttersprache				Gesamt	
	Gewünscht		Nicht gewünscht		Gewünscht		Nicht gewünscht		Gewünscht	Nicht gewünscht
	WÜ	SW	WÜ	SW	Deutsch	Andere	Deutsch	Andere		
Sport/Fitness/Bewegung	70	71	30	29	**68**	**79**	**32**	**21**	70	30
Psychischen Belastungen	70	68	30	32	68	76	32	24	69	31
Stressreduktion	68	60	32	40	64	60	36	40	65	35
Entspannung/Achtsamkeit	52	46	48	54	48	58	52	42	50	50
Prüfungsangst	**37**	**49**	**63**	**51**	**36**	**57**	**64**	**43**	41	59
Ernährung/gesund Kochen	44	49	56	51	44	50	56	50	46	54
Austausch mit anderen Studierenden	**42**	**28**	**58**	**72**	36	57	64	43	37	63
Resilienzförderung	37	29	63	71	36	27	64	73	34	66
Suchtentwöhnung	12	14	88	86	12	14	88	86	12	88

Angaben in Prozent (gerundet)

Signifikante Zusammenhänge (χ^2^) **fett** hervorgehoben

Für den Hochschulstandort zeigten sich signifikante Ergebnisse bei den Variablen Prüfungsangst (χ^2^[1] = 6,94; *p* = 0,008; φ = 0,11) und Austausch mit anderen Studierenden (χ^2^[1] = 9,43; *p* = 0,002; φ = −0,11). Während sich Studierende in SW vermehrt Angebote zum Thema Prüfungsangst wünschten, wurden Angebote in Form eines Austausches mit anderen Studierenden signifikant häufiger von Studenten und Studentinnen in WÜ gewünscht. Die jeweiligen Effektstärken, angegeben als φ‑Koeffizient, wiesen auf niedrige Effekte hin (r < 0,25; [8]).

Den Zusammenhang zwischen gewünschten gesundheitsförderlichen Angeboten und Muttersprache betrachtend, ließen sich statistisch signifikante Ergebnisse für die Variablen Sport/Fitness/Bewegung (χ^2^[1] = 14,17; *p* = 0,034; φ = 0,10) sowie Prüfungsangst (χ^2^[1] = 6,94; *p* < 0,001; φ = 0,17) feststellen. Auch wenn die Effektstärken erneut niedrig waren, zeigten die Daten, dass sich nicht-deutschsprachige Studierende signifikant häufiger als Deutsch-Muttersprachler:innen Sportangebote bzw. Angebote zur Bewältigung von Prüfungsangst wünschten.

### Kenntnisse über zielgruppenspezifische Gesundheitsangebote

Zuletzt war von Interesse, welche gesundheitsbezogenen und studierendenspezifischen Angebote den Studierenden an der FHWS bekannt sind und ob ein Zusammenhang zwischen der Bekanntheit und dem Hochschulstandort bzw. der Muttersprache besteht. Den Studierenden wurde eine Liste von sieben für FHWS-Studierende zugänglichen Einrichtungen präsentiert. Alle Angebote und Institutionen befanden sich in WÜ. Sie waren zwar ebenfalls für die Studierenden am Campus SW zuständig, jedoch nicht vor Ort vertreten. Zwei Drittel aller Befragten gaben an, bereits vom Hochschulsport gehört zu haben, die Psychotherapeutische Beratungsstelle des Studentenwerks war ca. einem Drittel der Teilnehmenden bekannt. Weniger bekannt waren nachfolgend die Sozialberatung des Studentenwerks (30 %), die Rechtsberatung des Studentenwerks (25 %), die Katholische Hochschulgemeinde (12 %), die Evangelische Studentengemeinde (10 %) sowie abschließend die Kontakt- und Informationsstelle für Studierende mit Behinderung und chronischer Erkrankung, welche nur 7 % der Befragten vertraut war. Es zeigte sich, dass – ausgenommen Hochschulsport – weniger als ein Drittel der FHWS-Studierenden über relevante Gesundheitsangebote Kenntnisse hatten. In Tab. 3 sind die Kenntnisse über existierende Angebote nach Hochschulstandort und Muttersprache getrennt dargestellt.

**Tab. 3:** Kenntnisse über Angebote differenziert nach Hochschulstandort und Muttersprache sowie die dazugehörigen statistischen Kennzahlen (Signifikanzwert *p*, Effektstärke Phi φ; Angaben in Prozent)

Angebote in Würzburg	Standort							Muttersprache							Gesamt	
	Bereits davon gehört		Noch nicht davon gehört		Statistik			Bereits davon gehört		Noch nicht davon gehört		Statistik			Bereits davon gehört	Noch nicht davon gehört
	WÜ	SW	WÜ	SW	χ^2^	p	φ	DEU^a^	Andere^b^	DEU	Andere	χ^2^	p	φ		
Hochschulsport	**77**	**45**	**23**	**55**	**52,87**	**<** **0,001**	**−0,33**	**75**	**35**	**25**	**65**	**59,46**	**<** **0,001**	**−0,35**	66	34
Psychotherapeutische Beratungsstelle des Studentenwerks	**39**	**18**	**61**	**82**	**24,57**	**<** **0,001**	**−0,22**	**37**	**13**	**63**	**87**	**20,36**	**<** **0,001**	**−0,20**	32	68
Sozialberatung des Studentenwerks	**35**	**20**	**65**	**80**	**12,36**	**<** **0,001**	**−0,16**	**33**	**22**	**67**	**78**	**4,26**	**0,039**	**−0,09**	30	70
Rechtsberatung des Studentenwerks	**28**	**18**	**72**	**82**	**5,63**	**0,018**	**−0,11**	26	20	74	80	1,62	0,203	−0,06	25	75
Katholische Hochschulgemeinde	**19**	**2**	**81**	**98**	**28,52**	**<** **0,001**	**−0,24**	**15**	**2**	**85**	**98**	**13,27**	**<** **0,001**	**−0,16**	13	87
Evangelische Studentengemeinde	**16**	**1**	**84**	**99**	**24,48**	**<** **0,001**	**−0,22**	**12**	**5**	**88**	**95**	**4,41**	**0,036**	**−0,09**	10	90
Kontakt- und Informationsstelle für Studierende mit Behinderung und chronischer Erkrankung	**9**	**4**	**91**	**96**	**4,48**	**0,034**	**−0,10**	7	5	93	95	0,82	0,364	−0,04	7	93

^a^DEU steht für Deutsch als Muttersprache

^b^Andere steht für andere Muttersprachen als Deutsch

Angaben in Prozent (gerundet)

Signifikante Zusammenhänge (χ^2^) **fett** hervorgehoben

Differenziert nach Hochschulstandort zeigten sich dementsprechend für alle sieben Angebote signifikante Unterschiede. Die Daten wiesen darauf hin, dass Studierende in SW von allen Angeboten signifikant weniger Kenntnis hatten. Für die Variable Hochschulsport lag ein mittlerer Effekt (r > 0,25) vor, während die weiteren Variablen niedrige Effektstärken aufwiesen [8]. Weiter zeigte sich bei fünf Angeboten ein signifikanter Zusammenhang zwischen Muttersprache und Bekanntheit. Diese fünf Anlaufstellen waren allesamt unter Deutschsprachler:innen bekannter als unter Fremdsprachler:innen. Auch hier war für die Variable Hochschulsport ein mittlerer Effekt (r > 0,25) feststellbar, während für die anderen Variablen niedrige Effektstärken und somit geringe Unterschiede vorlagen [8]. Zusätzlich wurde im Zuge der Umfrage mittels offener Angabe abgefragt, welche spezifischen Angebote die Studierenden am Hochschulstandort SW kennen. Nur 9 % der in SW Studierenden gaben ihnen bekannte Angebote, darunter v. a. den Hochschulsport, an. Da ein signifikanter Zusammenhang zwischen Hochschulstandort und Muttersprachlichkeit vorliegt (φ = 0,476; *p* < 0,001), wurde der Zusammenhang zwischen Angebotskenntnis sowie Muttersprache zudem getrennt für jeden Standort anhand des φ‑Koeffizienten berechnet. Für den Standort Würzburg ergaben sich signifikante Zusammenhänge zwischen Muttersprache und Kenntnis über die Psychotherapeutische Beratungsstelle (φ = −0,117; *p* = 0,036) sowie über den Hochschulsport (φ = −0,120; *p* = 0,031). Diese beiden Einrichtungen sind unter den Studierenden des Standorts Würzburg, die eine andere Muttersprache als Deutsch sprechen, signifikant weniger bekannt. Den Standort Schweinfurt betrachtend zeigte sich ein Zusammenhang zwischen Muttersprache und Hochschulsport (φ = −0,352; *p* < 0,001). Auch dort sind die internationalen Studierenden signifikant schlechter über dieses Angebot informiert als ihre deutschen Kommiliton:innen.

## Diskussion

Ziel der vorliegenden Studie war es zu untersuchen, welche Gesundheitsbereiche die Studierenden als bedeutsam für ihr Studium betrachten, welche Angebote sich Studierende an der FHWS wünschen, welche bereits bestehenden Angebote ihnen bekannt sind und, ob signifikante Unterschiede zwischen Hochschulstandort und Muttersprache hinsichtlich der untersuchten Variablen vorliegen.

Wie die vorliegende Erhebung gezeigt hat, sind besonders Stress und psychische Belastung für die befragten Studierenden an der FHWS von großer Bedeutung. Dieses Ergebnis steht im Einklang mit weiteren in Deutschland durchgeführten Studien [7, 27, 34]. Als Ursachen für die besondere psychische Belastung von Studierenden werden in der Literatur vielfältige, studienbezogene Schwierigkeiten diskutiert. Vor allem der sich anzueignende Stoffumfang wird von Studierenden oftmals als zu hoch beurteilt. 72 % von knapp 8000 Studierenden des HISBUS-Panels, die Probleme hinsichtlich dieser Thematik haben, gaben in der Untersuchung von Middendorf et al. [27] an, einen sehr starken Leistungsdruck im Studium zu erfahren. Andere Faktoren wie beispielsweise der notwendige Zeitaufwand für das Studium scheinen ebenso eine Rolle zu spielen. Ein hohes Stresserleben im Studium korreliert mit einer geringen Lebenszufriedenheit, häufigen psychosomatischen Beschwerden, schlechter selbstberichteter Gesundheit sowie ungesundem Ernährungsverhalten [31].

Sucht/Konsum wurde von 59 % der Befragten als nicht bedeutsam bezüglich des Studiums eingeschätzt, nur 5 % stuften diesen Gesundheitsbereich hingegen als sehr relevant ein. Im Rahmen der Sozialerhebung des Deutschen Studentenwerks gab 1 % der Befragten an, einen Beratungsbedarf bezüglich Alkohol- oder Drogenkonsum zu haben [26]. Dieser Bedarf weicht der Datenlage zufolge jedoch von der tatsächlichen Konsumprävalenz ab. Substanzkonsum zur Leistungssteigerung (Gehirndoping/Neuroenhancement) wird laut der Umfrage von Middendorf et al. [27] von 5 % der Studierenden praktiziert. Definiert wird Gehirndoping in diesem Fall als die gezielte Einnahme von illegalen Substanzen (Amphetamin, Cannabis etc.) oder von verschreibungspflichtigen Medikamenten (Schlafmittel, Methylphenidat, Antidepressiva etc.) mit dem Ziel der Leistungssteigerung. Diese Zahl steht in Übereinstimmung mit der Anzahl der Befragten in der vorliegenden Untersuchung, die Sucht/Konsum als sehr relevant einschätzten. Andere Studien [24, 30] dokumentieren mit Anteilen zwischen 12 und 16 % eine deutlich größere Prävalenz von studienbezogenem Gehirndoping. Betrachtet man den Konsum psychogener Substanzen aus anderen Gründen als zur Leistungssteigerung, steigen die Zahlen an und liegen teilweise deutlich über dem der gleichaltrigen Allgemeinbevölkerung [29]. In Bezug auf illegale Substanzen wird unter Studierenden am häufigsten zu Cannabis gegriffen. Die Zahlen schwanken zwischen 22 % (12-Monats-Prävalenz; [29]) und 34 % (3-Monats-Prävalenz; [24]) für Studierende der Sozialen Arbeit. Die Prävalenzen für legale Substanzen liegen erwartungsgemäß höher. Über die Hälfte der Studierenden wiesen laut der Studie von Ganz et al. [13] einen riskanten Alkoholkonsum im letzten Monat auf. Studierende mit riskantem Konsum zeigen mehr alkoholassoziierte Probleme, die das Studium beeinträchtigen können als diejenigen mit geringem Konsum [13]. Die vorliegende Diskrepanz zwischen teilweise hohem Konsum und relativ geringer Relevanz des Konsums in Bezug auf das Studium, lässt sich u. U. damit erklären, dass den Studierenden die Auswirkungen von beispielsweise Alkohol auf ihr Studium nicht bewusst sind oder dass sie die Folgen des Substanzgebrauchs unterschätzen. Auch unter den Studierenden der FHWS kann zuweilen, aufgrund der geringen Relevanzeinschätzung des Bereichs Sucht/Konsum, eine solche Widersprüchlichkeit angenommen werden.

Zwei der abgefragten Gesundheitsbereiche, nämlich Prüfungsangst und körperliche Beschwerden, wurden im Vergleich zu den Würzburger Student:innen von den Studierenden am Standort SW als bedeutsamer eingeschätzt. In SW sind primär technische Studiengänge angesiedelt, die mehrheitlich von Studenten absolviert werden. Die Durchfallquote liegt in diesen Fächern meist über dem Durchschnitt. 2014 wurden in Deutschland durchschnittlich 41 von 1000 Abschlussprüfungen nicht bestanden. Die Fächer Chemieingenieurwesen (124 je 1000), Energietechnik (118 je 1000), Werkstoffwissenschaft (117 je 1000) und Mechatronik (109 je 1000) wiesen hohe Durchfallquoten auf, während im Fach Soziale Arbeit nur 4 von 1000 Abschlussprüfungen nicht bestanden wurden. Einhergehend mit der Fächerwahl ist die Durchfallquote der Männer (57 je 1000) mehr als doppelt so hoch als die der Frauen (25 je 1000; [14]). Diese standortspezifischen Umstände könnten den Unterschied zwischen SW und WÜ hinsichtlich der Variable Prüfungsangst begründen. Als Erklärung für die größere Bedeutung des Themas körperliche Beschwerden für Studierende in SW dient möglicherweise das limitierte Hochschulsportangebot in SW. Während im Sommersemester 2022 dort lediglich drei Kurse angeboten wurden, gab es in WÜ ein vielfältiges und umfangreiches Kursangebot über den dort ansässigen Hochschulsport.

In Deutschland waren im Wintersemester 2020/2021 12 % ausländische Studierende eingeschrieben [32]. Deutsch- und nicht-deutschmuttersprachige Studierende unterscheiden sich laut der vorliegenden Erhebung in vier Gesundheitsbereichen signifikant voneinander. So schätzten Studierende mit einer anderen Muttersprache als Deutsch die Bereiche Prüfungsangst, körperliche Beschwerden/Krankheit, Ernährung und Sucht/Konsum als signifikant relevanter ein als ihre deutschsprachigen Kommilitonen und Kommilitoninnen. Dies könnte möglicherweise daran liegen, dass internationale Studierende insgesamt stärker von psychischen Störungen (z. B. Depressionen) betroffen sind, sie größere Schwierigkeiten mit dem Verständnis des Gesundheitssystems vor Ort haben und sie weniger psychologische Hilfe in Anspruch nehmen als einheimische Kommilitonen und Kommilitoninnen [1, 6]. Knapp die Hälfte von 67.000 befragten Studierenden (20 % mit Migrationshintergrund, jedoch keine Bildungsausländer:innen) mit Beratungsbedarf suchten eine passende Unterstützung auf [26]. Bei einer Stichprobe von internationalen Studierenden nahmen nur 20 % der Befragten mit Beratungsbedarf den Beratungsservice der Universität in Anspruch [28]. Für die Variable Prüfungsangst konnte der höchste Effekt festgestellt werden. Der Erfolg des Studiums ist für internationale Studierende gemäß § 16 Aufenthaltsgesetz (AufenthG) meist direkt verknüpft mit der Aufenthaltserlaubnis. So erteilt die Ausländerbehörde bisweilen keine Verlängerung, wenn keine positive Prognose für einen Studienerfolg in angemessener Zeit zu erwarten ist (vgl. § 16 Abs. 2 AufenthG). Dies liegt mitunter vor, wenn Studierende nach einer gewissen Anzahl an Semestern zu wenige ECTS-Punkte (European Credit Transfer and Accumulation System) erreicht haben. Ob die Prognose positiv oder negativ bewertet wird, hängt maßgeblich von bisher erbrachten Leistungsnachweisen ab, die bei jeder beantragten Verlängerung geprüft werden können [12]. Der Umstand, dass die Aufenthaltserlaubnis an ein erfolgreiches Studium geknüpft ist, führt mutmaßlich zu erhöhter Prüfungsangst bei ausländischen Studierenden. Zusätzlich belegen die internationalen Studierenden vermehrt technische Studiengänge, insbesondere in der Fächergruppe Ingenieurswissenschaften, in welchen hohe Durchfallquoten herrschen, was das Ergebnis ebenfalls erklären könnte [33]. Ferner können auch sprachliche Barrieren erhöhte Prüfungsangst begründen, da viele internationale Studierende das Studium in einer anderen als in ihrer Muttersprache absolvieren und somit auch Prüfungen in Englisch oder Deutsch ablegen müssen.

Betrachtet man die Wunschangebote so ist nachvollziehbar, dass die Studierenden am Standort in SW sowie die nicht-deutschmuttersprachigen Studierenden sich signifikant häufiger Angebote zum Thema Prüfungsangst wünschen, da diese Personengruppen stärker von dieser Problematik betroffen sind. Die Analysen ergaben bei den internationalen Studierenden ferner eine größere Beeinträchtigung durch körperliche Beschwerden/Krankheit, weshalb ebenfalls naheliegend ist, dass diese sich häufiger Sport- und Bewegungsangebote wünschen als ihre deutschsprachigen Kommiliton:innen. Befragte aus Afrika und Asien, die in den USA studieren, verbrachten laut einer Studie weniger Zeit mit physischen Aktivitäten als Studierende von anderen Kontinenten [36]. Auch an der FHWS stammt ein Großteil der ausländischen Studierenden aus Afrika und Asien. Die Vergleichbarkeit der Ergebnisse kann jedoch aufgrund der Datenerhebung in den USA und in Deutschland eingeschränkt sein. Weiter zeigt die Umfrage, dass FHWS-Student:innen wichtige Angebote und Anlaufstellen kaum oder überhaupt nicht bekannt sind. Lediglich vom Hochschulsport haben 77 % der Würzburger Studentinnen und Studenten bereits gehört, alle anderen Angebote sind mehr als der Hälfte der Befragten nicht geläufig. Dies deckt sich mit den Ergebnissen von Klemmt et al. [23]. Die Forschenden fanden heraus, dass es zwar zahlreiche Angebote für Studierende in WÜ gibt, diese zuweilen jedoch nicht wahrgenommen werden. Da sich die hier untersuchten Anlaufstellen allesamt in WÜ befinden, lässt sich begründen, weshalb die Bekanntheit der Angebote sich signifikant unter den Studierenden der beiden Standorte unterscheidet. Wenn auch Studierende in WÜ mitunter schlecht informiert scheinen, zeigen die Ergebnisse, dass die Institutionen unter den Schweinfurter Studierenden deutlich unbekannter sind, trotz der Tatsache, dass diese ebenfalls für diese Personengruppe zuständig sind. Dies betont die Notwendigkeit Studierende von Hochschulstandorten, die eine erhöhte räumliche Distanz zu relevanten gesundheitsförderlichen Einrichtungen aufweisen, vermehrt zu informieren, um Nutzungsbarrieren abzubauen. Auch nicht-deutschmuttersprachige Studierende sind wenig über Angebote und Anlaufstellen im Bilde, was damit erklärt werden kann, dass diese ebenfalls häufig am Standort in SW studieren und viele Informationsquellen (z. B. Homepages, Flyer etc.) nur auf Deutsch vorhanden sind. Die höhere Relevanz von vier der sieben abgefragten Gesundheitsbereiche für internationale Studierende legt nahe, dass diese z. T. einen höheren Unterstützungsbedarf haben. Diese Personengruppe sollte demnach vermehrt in den Blick genommen werden. Gesundheitliche Aufklärung und Information sollte zudem ebenfalls auf Englisch oder bilingual angeboten werden.

Studien legen den Zusammenhang zwischen Gesundheit, Gesundheitsverhalten sowie Bildungserfolg nahe und betonen die Bedeutsamkeit von Gesundheitsförderung an Hochschulen [11]. Dies sollte sowohl auf der Ebene der Verhältnisprävention (strukturelle Prävention) als auch auf der Ebene der Verhaltensprävention (individuelle Prävention) ansetzen. Änderungen im Curriculum und der Studienorganisation scheinen effektive verhältnispräventive Maßnahmen darzustellen [25]. Empirische Evidenzen liegen zudem für wirksame verhaltenspräventive Interventionen an der Hochschule vor. Stressmanagementkurse können sich beispielsweise positiv auf das Stresserleben und Depressions- und Angstsymptome bei Studierenden auswirken [3]. Auch für Kurse, die einen gesunden Lebensstil bei Studierenden fördern, liegen Wirksamkeitsnachweise vor [2]. Hochschulen stellen somit ein adäquates Setting der Gesundheitsförderung dar [23]. Durch das 2015 erlassene Präventionsgesetz sind die Krankenkassen gesetzlich verpflichtet gesundheitsförderliche Maßnahmen in den Hochschulen und insbesondere für Studierende inhaltlich und finanziell zu unterstützen (vgl. § 20 SGB V PrävG; [21]). Eine innerhalb der Hochschulen implementierte und von den gesetzlichen Krankenkassen finanzierte Gesundheitsförderung kann somit eine effektive und ökonomische Möglichkeit darstellen, zielgruppenspezifische Prävention auf struktureller und individueller Ebene umzusetzen.

Die vorliegende Untersuchung zeigt Bedarfe, Wünsche und Angebotskenntnisse der Studierenden an der FHWS auf. Diese Erkenntnisse können und sollen im Rahmen des Studentischen Gesundheitsmanagements weiter genutzt werden.

Die durchgeführte Erhebung weist einige Limitationen auf. Zuerst muss angemerkt werden, dass die Stichprobe einen Zusammenhang zwischen Standort und Muttersprache aufweist. Es sind prozentual mehr internationale Studierende am Standort in Schweinfurt eingeschrieben, was bei der Interpretation der Daten berücksichtigt werden muss. Des Weiteren bezieht sich die Stichprobe nur auf Studierende der FHWS, weshalb die Repräsentativität eingeschränkt sein kann, auch wenn die Ergebnisse Vergleichbarkeiten zu anderen Studien aufweisen. Zudem handelt es sich um eine Querschnittstudie, die lediglich Zusammenhänge jedoch keine Kausalitäten aufzeigen kann und nur eine Momentaufnahme der aktuellen Situation abbildet. Bedacht werden muss außerdem ein möglicher Einfluss der Coronapandemie auf die Ergebnisse, die vermutlich vermehrt die internationalen Studierenden belastet hat.

## Fazit für die Praxis

- Angebote in den Bereichen Stress und psychische Belastung sollten vorrangig an der Hochschule implementiert werden, da diese Bereiche als am relevantesten eingestuft wurden und zudem, gemeinsam mit Bewegungs- und Sportangeboten, von den Studierenden am häufigsten gewünscht wurden.
- Nicht-deutschmuttersprachige Studierende sowie Studierende am Standort in Schweinfurt (SW) schätzen einige Gesundheitsbereiche als relevanter für ihr Studium ein als andere Studierende. Dies lässt darauf schließen, dass diese Studierendengruppen besonders von Gesundheitsangeboten und Unterstützung profitieren könnten.
- Wesentliche studierendenspezifischen Gesundheitsangebote sind den Studierenden mitunter kaum bekannt. Vor allem Studierende in SW und internationale Studierende haben wenig Kenntnisse über bereits existierende Angebote. Die Studierenden sollten mehr Informationen über erreichbare Unterstützungsmöglichkeiten erhalten. Die Informationsverbreitung sollte außerdem vermehrt am Campus in SW und in englischer Sprache erfolgen.
